# Prediction of Advanced Axillary Lymph Node Metastases (ypN2-3) Using Breast MR imaging and PET/CT after Neoadjuvant Chemotherapy in Invasive Ductal Carcinoma Patients

**DOI:** 10.1038/s41598-018-21554-z

**Published:** 2018-02-16

**Authors:** Won Hwa Kim, Sang-Woo Lee, Hye Jung Kim, Yee Soo Chae, Shin Young Jeong, Jin Hyang Jung, Ho Yong Park, Won Kee Lee

**Affiliations:** 1 Department of Radiology, Kyungpook National University Chilgok Hospital, Daegu, Republic of Korea; 2 Department of Radiology, School of Medicine, Kyungpook National University, Daegu, Republic of Korea; 3 Department of Nuclear Medicine, Kyungpook National University Chilgok Hospital, Daegu, Republic of Korea; 4 Department of Nuclear Medicine, School of Medicine, Kyungpook National University, Daegu, Republic of Korea; 5 Departments of Oncology/Hematology, Kyungpook National University Chilgok Hospital, Daegu, Republic of Korea; 6 Departments of Oncology/Hematology, School of Medicine, Kyungpook National University, Daegu, Republic of Korea; 7 Department of Surgery, Kyungpook National University Chilgok Hospital, Daegu, Republic of Korea; 8 Department of Surgery, School of Medicine, Kyungpook National University, Daegu, Republic of Korea; 9 Center of Biostatistics, School of Medicine, Kyungpook National University, Daegu, Republic of Korea

## Abstract

We aimed to investigate the value of breast magnetic resonance (MR) imaging and positron emission tomography-computed tomography (PET/CT) in predicting advanced axillary lymph node (ALN) metastases (ypN2-3) after neoadjuvant chemotherapy (NAC) in invasive ductal carcinoma patients. A total of 108 patients with invasive ductal carcinoma underwent breast MR imaging and PET/CT both before and after NAC (termed initial staging and restaging, respectively). The number of positive ALNs and the short diameter (SD) of the largest ALN on breast MR imaging and maximal standardized uptake value (SUVmax) in the ALNs on PET/CT were evaluated. Odds ratio (OR) for prediction of advanced ALN metastases was calculated. The negative predictive value (NPV) of restaging imaging for exclusion of advanced ALN metastases was also calculated. Patients with advanced ALN metastases were more likely to have a higher number (≥2) of positive LNs (OR, 8.06; *P* = 0.015) on restaging MR imaging. No clinico-pathological factors were significantly associated with advanced ALN metastases. With restaging MR imaging, PET/CT, and MR imaging plus PET/CT, the NPV for excluding advanced ALN metastases was 97.3%, 94.4%, and 100.0%. A higher number of positive ALNs on restaging MR imaging was an independent predictor for advanced ALN metastases after NAC.

## Introduction

The evaluation of axillary lymph node (ALN) metastases is a crucial step in patients with breast cancer to guide locoregional and systemic treatment decisions. Pathological nodal staging is determined by the number of metastatic ALNs, and advanced ALN metastases (pN2-3) is defined as having more than three metastatic ALNs. For the evaluation of nodal metastases, sentinel lymph node biopsy (SLNB) is the standard surgical technique for less-invasive approach^1,2^. If metastatic LNs were not found in SLNB, further axillary lymph node dissection (ALND) is generally not required. This is also accepted even when metastatic LNs were found in SLNB but confined to the one or two metastatic LNs, according to the American College of Surgeons Oncology Group (ACOSOG) Z1011 trial, as ALND offers no significant diagnostic benefit and increases morbidity^3^. Therefore, attention has been redirected toward the prediction of advanced ALN metastases with the endeavor of differentiating between non-advanced and advanced ALN metastases^4,5^.

Currently, the less-invasive approach of axillary surgery is also increasingly being considered for patients with breast cancer undergoing neoadjuvant chemotherapy (NAC), which reduces tumor burden in the axilla, resulting in the negative conversion of nodal metastases at rates between 23% and 41%^6–8^. Multiple studies, including ACOSOG Z1071 and SENTINA (Sentinel Neoadjuvant), have demonstrated the feasibility of SLNB after NAC^8,9^. In those studies, they found that SLNB was generally feasible with a high success rate (80–99%); however, false-negative rate (FNR) of SLNB is still problematic. Axillary restaging using ultrasound after NAC is one of the strategies to decrease FNR of SLNB^10^. However, there still is a controversy regarding the role of imaging in predicting nodal status after NAC^11,12^. Furthermore, the diagnostic performance of breast magnetic resonance (MR) imaging and positron emission tomography-computed tomography (PET/CT) in predicting advanced ALN metastases has not been fully evaluated.

Therefore, the aim of this study was to investigate the value of breast MR imaging and PET/CT in predicting advanced ALN metastases after NAC in patients with invasive ductal carcinoma. In addition, we evaluated the diagnostic performance of single and combined use of breast MR imaging and PET/CT.

## Materials and Methods

### Study Population

This study was approved by our institutional review board and the requirement for informed consent was waived. From a review of the breast-imaging database of Kyungpook National University Chilgok Hospital, we identified 139 consecutive patients with invasive ductal carcinoma who underwent NAC between January 2011 and December 2014. At our institution, patients with clinical stages of T2 or greater and/or patients with clinical stages of N1 or greater are potentially eligible for NAC treatment. Clinical nodal staging was performed via imaging studies (ultrasound, MR imaging, and PET/CT), as well as via fine-needle aspiration biopsy, and physical examinations. Patients underwent breast MR imaging and PET/CT prior to initiation of NAC treatment (initial staging) and after completion of NAC treatment but before surgery (restaging). Among these patients, we excluded those who had no breast MR imaging or PET/CT at initial staging or restaging (n = 29) and/or with excisional biopsy at initial presentation (n = 2). Finally, 108 women who had breast MR imaging and PET/CT at initial staging and restaging after NAC were enrolled in this study. NAC was given with a taxane-based regimen consisting of doxorubicin and cyclophosphamide followed by docetaxel. Patients with human epidermal growth factor receptor 2 (HER2) were additionally treated with trastuzumab.

### Image Acquisition

Breast MR imaging was performed using a 3.0-T system (Discovery MR750, GE Healthcare) with a dedicated eight-channel surface breast coil. Each patient received an intravenous injection of 0.1 mL/kg of gadobutrol (Gadovist, Bayer Schering Pharma, Berlin, Germany) as the contrast agent. Axial T1-weighted images (TR/TE, 699/10.5; matrix, 352 × 256; slice thickness, 3 mm) and axial fat-suppressed, T2-weighted images (TR/TE, 8353/90; matrix, 384 × 256; slice thickness, 3 mm) were acquired. Dynamic contrast-enhanced MR examination included one precontrast and five postcontrast imaging with bilateral axial acquisition using fat-suppressed T1-weighted imaging. Subtraction images and three-dimensional maximum intensity projection (MIP) images were generated for all studies.

PET/CT for whole-body scanning was performed after patients had fasted for at least 6 hours using the following PET/CT systems: Discovery 600 and Discovery 690 (GE Healthcare, Milwaukee, WI, USA). Approximately 3.7–5.6 MBq of ^18^F-fluorodeoxyglucose (18F-FDG) per kilogram of body weight was injected intravenously (IV), and the patients were advised to rest for 1 hour before image acquisition. For attenuation correction, a low-dose CT scan was obtained before the PET scan without contrast enhancement from the base of the skull vertex to the knee. The PET scan was performed with a maximal space resolution of 5.1 mm (Discovery 600) and 4.9 mm (Discovery 690) at 1.5 minute per bed position.

### Image Analysis

For breast MR imaging, two breast-imaging radiologists (H.J.K. and W.H.K., with 18 years and 10 years of experience, respectively) determined the number of positive ALNs and measured the short diameter (SD) of the largest ALN from the ipsilateral breast in the images. The ALN was considered to be positive when one or more findings were noted as follows: cortical thickness >3 mm, existence of eccentric cortical thickening, loss of fatty hilum, and round or lobulated nodal shape^10,13–16^. The ALNs ipsilateral to the breast cancer were compared to the contralateral axillary LNs. If the ALNs did not show any significant difference in the above characteristics, the ALNs were considered negative, which is in line with previous studies^15,17^.

For PET/CT, one nuclear medicine physician (S.W.L. with 13 years of experience) and one breast-imaging radiologist (W.H.K.) reviewed the images in consensus. Regions of interest (ROI) were manually placed over the area of highest activity on slices of the ALNs, and the maximal standardized uptake value (SUVmax) within the ROI was obtained. Especially in the ALNs of low ^18^F-FDG uptake (SUVmax <1), the ROI was carefully drawn over the corresponding ALNs on CT images. The SUVmax was calculated using the volume viewer software on a GE Advantage Workstation 4.6 (GE Healthcare) with the following formulas: SUVmax = maximum activity in ROI (MBq/g)/[injected dose (MBq)/body weight (g)]. For both breast MR imaging and PET/CT, all reviewers knew that the patients had invasive breast cancer; however, no further information, including pathological nodal stages, was given to the reviewers.

### Pathological Evaluation

All patients underwent axillary surgery with SLNB and/or ALND, and the final pathological nodal stage was determined based on the histopathological results of surgical specimens. ALNs were examined using hematoxylin and eosin staining. Each ALN was classified as negative or positive for metastases; the total numbers of ALNs sampled and the number with metastases were recorded. All histopathological evaluation was performed by a pathologist (J.Y.P. with 18 years of experience in breast pathology).

### Data Collection and Statistical Analysis

Clinical data included age, as well as clinical tumor (T), and clinical lymph nodes (N) stages. The following pathological information from percutaneous biopsy results obtained before NAC were included: histological grade, estrogen receptor (ER), progesterone receptor (PR), and HER2 status. Clinical T and N staging was performed at initial diagnosis on the basis of the 7th American Joint Committee on Cancer^18^. The expression of ER, PR, and HER2 was assessed by immunohistochemical staining. The expression of ER and PR was quantified using the Allred score, considering a total Allred score >2 as positive for ER or PR^19^. A HER2 score of 0 or 1 was considered negative (HER2-negative), a value of 3 was considered positive (HER2-positive), and value of 2 was considered equivocal. For equivocal cases, silver-enhanced *in situ* hybridization (SISH) was performed and an HER2/CEP17 ratio ≥2 or an HER2/CEP17 ratio <2 with an average HER2 copy number ≥6 were considered positive (HER2-positive)^20^. Hormone receptor (HR)-positive status was defined as tumors expressing ER and/or PR.

The optimal cut-off values of the number of positive ALNs or SD of the largest ALN on breast MR imaging, and the nodal SUVmax on PET/CT were independently calculated from receiver operating characteristic (ROC) analysis, using the best Youden index (sensitivity + specificity − 1) for the prediction of residual or advanced ALN metastases. Then, patients were divided into binary groups according to each cut-off value. Diagnostic performances of restaging MR imaging or PET/CT, including overall diagnostic accuracy, sensitivity, specificity, positive predictive value (PPV), and negative predictive value (NPV), were estimated for each modality, and a combination of both modalities (restaging MR imaging plus PET/CT) for the prediction of advanced ALN metastases. For restaging MR imaging, positive was defined either as having either the number of positive ALNs or as having the SD of the largest ALN above the cut-off value. For restaging MR imaging plus PET/CT, positive was defined when either modality was above the cut-off value. The overall diagnostic accuracy was estimated by calculating the area under the ROC curve (AUC) with 95% confidence interval (CI). Sensitivity was compared using the McNemar’s test, and the comparison of the AUC was performed with the method of Delong *et al*.^21^.

The clinico-pathological and imaging findings of patients with and without residual or advanced ALN metastases were compared using the independent *t* test or chi-square test as appropriate. The OR (odds ratio) and 95% CI for residual or advanced ALN metastases were calculated with univariate logistic regression analysis, and variables with *P* < 0.10 were selected for the final multivariate model. All statistical analyses were performed with the statistical software SPSS version 24.0 (Chicago, IL, USA) and MedCalc version 17.1 (Mariakerke, Belgium). Two-tailed *P* values of less than 0.05 were considered statistically significant.

### Ethical Standards

All procedures performed in studies involving human participants were in accordance with the ethical standards of the institutional and/or national research committee and with the 1964 Helsinki declaration and its later amendments or comparable ethical standards. Due to our retrospective review of the data, and the requirement of an informed consent was waived after approval of institutional review board.

## Results

The mean age of the patients was 46.4 years (range, 27–64 years). At initial staging, 13 patients (12.0%) had clinical T stage 1 (cT1), 68 (63.0%) had cT2, 21 (19.4%) had cT3, and 6 (5.6%) had cT4. Regarding clinical N stages (cN), 6 patients (5.6%) had cN0, 42 (38.9%) had cN1, 45 (41.7%) had cN2, and 15 (13.9%) had cN3. Of the 102 patients with cN1-N3, 78 patients (76.5%) had biopsy-proven nodal disease at initial diagnosis. Of all patients, 63 patients (58.3%) had no positive ALNs at MR imaging after NAC and 44 patients (40.7%) had positive ALNs with 1 ALN in 28 patients (25.9%), 2 ALNs in nine patients (8.3%), 3 ALNs in two patients (2.8%), and 4 ALNs in five patients (4.6%). For the surgical staging, 21 patients (19.4%) underwent SLNB only, 58 (53.7%) underwent ALND only, and the remaining 29 (26.9%) underwent both SLNB and ALND. The median number of ALNs excised at SLNB or ALND was 10 (range, 1–31). At the final pathological staging, 49 patients (45.4%) had residual ALN metastases, including 12 patients (11.1%) with advanced ALN metastases ranging in number from 5 to 15. Among the patients with initial node-positive disease (cN1 or greater), 55 (53.9%) patients had negative ALNs at the final pathological nodal staging.

### Factors Associated with Residual ALN metastases (ypN1-3)

Tables 1 and 2 summarize the factors associated with residual ALN metastases (ypN1-3) following NAC. Based on univariate analysis, factors associated with residual ALN metastases were HR status (*P* = 0.011), HER2 status (*P* = 0.046), the number of positive ALNs at initial and restaging MR imaging (*P* = 0.032 and *P* = 0.001, respectively), SD of the largest ALN at initial MR imaging (*P* = 0.015), and the nodal SUVmax at restaging PET/CT (*P* = 0.012). Nodal SUVmax at initial staging PET/CT was associated with residual ALN metastases with a borderline significance (*P* = 0.053). Based on multivariate analysis, HR status (*P* = 0.028) and nodal SUVmax at restaging PET/CT (*P* = 0.020) were independently associated with residual ALN metastases. Patients with residual ALN metastases were more likely to have a positive HR status (OR at multivariate analysis, 3.07; 95% CI: 1.13, 8.33) and higher (>0.9) nodal SUVmax (OR, 3.72; 95% CI: 1.24, 11.23) at restaging PET/CT.

**Table 1:** Imaging and Clinico-Pathological Factors between Patients with and without Residual ALN metastases after Neoadjuvant Chemotherapy.

Characteristics	Patients without Residual ALN metastases (n = 59)	Patients with Residual ALN metastases (n = 49)	*P* Value
Age, years*	45.2 ± 8.3	47.8 ± 8.5	0.110
Initial clinical T stages			0.425
T1	7 (11.9%)	6 (12.2%)	
T2	39 (66.1%)	29 (59.2%)	
T3	11 (18.6%)	10 (20.4%)	
T4	2 (3.4%)	4 (8.2%)	
Initial clinical N stages			0.162
N0	4 (6.8%)	2 (4.1%)	
N1	26 (44.1%)	16 (32.7%)	
N2	22 (37.3%)	23 (46.9%)	
N3	7 (11.9%)	8 (16.3%)	
Histologic grade			0.208
Low to moderate	42 (71.2%)	40 (81.6%)	
High	17 (28.8%)	9 (18.4)	
HR status			0.011
Negative	30 (50.8%)	13 (26.5%)	
Positive	29 (49.2%)	36 (73.5%)	
HER2 status			0.046
Negative	31 (47.0%)	35 (71.4%)	
Positive	28 (66.7%)	14 (28.6%)	
Number of positive ALNs on initial staging MR imaging**			0.032
<7	51 (86.4%)	34 (69.4%)	
≥7	8 (13.6%)	15 (30.6%)	
Number of positive ALNs on restaging MR imaging**			0.001
0	43 (72.9%)	20 (40.8%)	
≥1	16 (27.1%)	29 (59.2%)	
Short diameter of the largest ALN on initial staging MR imaging			0.015
≤18.0 mm	56 (94.9%)	39 (79.6%)	
>18.0 mm	3 (5.1%)	10 (20.4%)	
Short diameter of the largest ALN on restaging MR imaging			0.296
≤6.8 mm	44 (74.6%)	32 (65.3%)	
>6.8 mm	15 (25.4%)	17 (34.7%)	
Nodal SUVmax on initial staging PET/CT			0.053
≤6.4	42 (71.2%)	26 (53.1%)	
>6.4	17 (28.8%)	23 (46.9%)	
Nodal SUVmax on restaging PET/CT			0.012
≤0.9	59 (100%)	44 (89.8%)	
>0.9	0	5 (10.2%)	

HR = hormone receptor; HER2 = human epidermal growth factor receptor 2; ALN = axillary lymph node; SUVmax = maximal standardized uptake value.

Data are the numbers of patients, with percentages in parentheses unless otherwise indicated by an asterisk.

*Data are mean ± standard deviation.

**The positive axillary LN was considered when one or more findings were noted as follows: cortical thickness >3 mm, eccentric cortical thickening, loss of fatty hilum, and round or lobulated nodal shape at MR imaging.

**Table 2:** Univariate and Multivariate Analyses Associated with Residual ALN metastases after Neoadjuvant Chemotherapy.

Characteristics	Univariate OR (95% CI)	Multivariate OR (95% CI)	*P* value
HR status			
Negative	1.00	1.00	
Positive	2.86 (1.27, 6.47)	3.07 (1.13, 8.33)	0.028
HER2 status			
Negative	1.00	1.00	
Positive	0.44 (0.20, 0.99)	0.48 (0.19 1.22)	0.125
Number of positive ALNs on initial staging MR imaging*			
<7	1.00	1.00	
≥7	2.81 (1.08, 7.36)	2.63 (0.83, 8.37)	0.102
Number of positive ALNs on restaging MR imaging*			
0	1.00	1.00	
≥1	3.90 (1.74, 8.75)	1.96 (0.76, 5.07)	0.164
Short diameter of the largest ALN on initial staging MR imaging			
≤18.0 mm	1.00	1.00	
>18.0 mm	4.79 (1.24, 18.53)	2.77 (0.56, 13.62)	0.211
Nodal SUVmax on initial staging PET/CT			
≤6.4	1.00	1.00	
>6.4	2.19 (0.99, 4.84)	1.00 (0.37, 2.67)	0.996
Nodal SUVmax on restaging PET/CT			
≤0.9	1.00	1.00	
>0.9	3.10 (1.27, 7.56)	3.72 (1.24, 11.23)	0.020

HR = hormone receptor; HER2 = human epidermal growth factor receptor 2; ALN = axillary lymph node; SUVmax = maximal standardized uptake value; OR = odds ratio; CI = confidence interval.

*The positive axillary LN was considered when one or more findings were noted as follows: cortical thickness >3 mm, eccentric cortical thickening, loss of fatty hilum, and round or lobulated nodal shape at MR imaging.

### Factors Associated with Advanced ALN metastases (ypN2-3)

Tables 3 and 4 summarize the factors associated with advanced ALN metastases (ypN2-3) after NAC. Based on univariate analysis, factors associated with advanced ALN metastases were the number of positive ALNs at initial and restaging MR imaging (*P* = 0.015 and *P* < 0.001, respectively), SD of the largest LN at restaging MR imaging (*P* = 0.007), and the nodal SUVmax at restaging PET/CT (*P* < 0.001). Nodal SUVmax at initial staging PET/CT was associated with advanced ALN metastases with a borderline significance (*P* = 0.060). Other clinico-pathological factors, including HR and HER2 statuses, were not significantly associated with advanced ALN metastases. According to multivariate analysis, higher (≥2) number of positive ALNs at restaging MR imaging (OR, 8.06; 95% CI: 1.51, 43.02, *P* = 0.015) was independently associated with advanced ALN metastases. A higher (≥4) number of positive ALNs at initial staging MR imaging (OR, 6.04; 95% CI: 0.81, 45.17, *P* = 0.080), a greater (>7.7 mm) SD of the largest ALN at restaging MR imaging (OR, 4.44; 95% CI: 0.92, 21.37, *P* = 0.063), and a higher (>1.1) nodal SUVmax at restaging PET/CT (OR, 4.56, 95% CI: 0.87, 23.81, *P* = 0.072) were associated with advanced ALN metastases with borderline significances.

**Table 3:** Imaging and Clinico-Pathological Factors between Patients with and without Advanced ALN metastases after Neoadjuvant Chemotherapy.

Characteristics	Patients without Advanced ALN metastases (n = 96)	Patients with Advanced ALN metastases (n = 12)	*P* Value
Age, years*	46.2 ± 8.5	48.3 ± 7.9	0.421
Initial clinical T stages			0.230
T1	13 (13.5%)	0	
T2	60 (62.5%)	8 (66.7%)	
T3	18 (18.8%)	3 (25.0%)	
T4	5 (5.2%)	1 (8.3%)	
Initial clinical N stages			0.195
N0	6 (6.2%)	0	
N1	38 (39.6%)	4 (33.3%)	
N2	40 (41.7%)	5 (41.7%)	
N3	12 (12.5%)	3 (25.0%)	
Histologic grade			0.937
Low to moderate	73 (76.0%)	9 (75.0%)	
High	23 (24.0%)	3 (25.0%)	
HR status			0.890
Negative	38 (39.6%)	5 (41.7%)	
Positive	58 (60.4%)	7 (58.3%)	
HER2 status			0.677
Negative	58 (60.4%)	8 (66.7%)	
Positive	38 (39.6%)	4 (33.3%)	
Number of positive ALNs on initial staging MR imaging**			0.015
<4	52 (54.2%)	2 (16.7%)	
≥4	44 (45.8%)	10 (83.3%)	
Number of positive ALNs on restaging MR imaging**			<0.001
<2	86 (89.6%)	5 (41.7%)	
≥2	10 (10.4%)	7 (58.3%)	
Short diameter of the largest ALN on initial staging MR imaging			0.295
≤ 7.7 mm	26 (27.1%)	5 (41.7%)	
>7.7 mm	70 (72.9%)	7 (58.3%)	
Short diameter of the largest ALN on restaging MR imaging			0.007
≤7.7 mm	80 (83.3%)	6 (50.0%)	
>7.7 mm	16 (16.7%)	6 (50.0%)	
Nodal SUVmax on initial staging PET/CT			0.060
≤2.6	34 (35.4%)	1 (8.3%)	
>2.6	62 (64.6%)	11 (91.7%)	
Nodal SUVmax on restaging PET/CT			<0.001
≤1.1	84 (87.5%)	5 (41.7%)	
>1.1	12 (12.5%)	7 (58.3%)	

HR = hormone receptor; HER2 = human epidermal growth factor receptor 2; ALN = axillary lymph node; SUVmax = maximal standardized uptake value.

Data are numbers of patients, with percentages in parentheses unless otherwise indicated by an asterisk.

*Data are mean ± standard deviation.

**The positive axillary LN was considered when one or more findings were noted as follows: cortical thickness>3 mm, eccentric cortical thickening, loss of fatty hilum, and round or lobulated nodal shape at MR imaging.

**Table 4:** Univariate and Multivariate Analyses Associated with Advanced ALN metastases after Neoadjuvant Chemotherapy.

Characteristics	Univariate OR (95% CI)	Multivariate OR (95% CI)	*P* value
Number of positive ALNs on initial staging MR imaging*			
<4	1.00	1.00	
≥4	5.91 (1.23, 28.41)	6.04 (0.81, 45.17)	0.080
Number of positive ALNs on restaging MR imaging*			
<2	1.00	1.00	
≥2	12.04 (3.21, 45.13)	8.06 (1.51, 43.02)	0.015
Short diameter of the largest ALN on restaging MR imaging			
≤ 7.7 mm	1.00	1.00	
>7.7 mm	5.00 (1.43, 17.49)	4.44 (0.92, 21.37)	0.063
Nodal SUVmax on initial staging PET/CT			
≤2.6	1.00	1.00	
>2.6	6.03 (0.75, 48.74)	3.06 (0.24, 39.17)	0.391
Nodal SUVmax on restaging PET/CT			
≤1.1	1.00	1.00	
>1.1	9.80 (2.68, 35.86)	4.56 (0.87, 23.81)	0.072

HR = hormone receptor; HER2 = human epidermal growth factor receptor 2; LN = lymph node; SUVmax = maximal standardized uptake value; OR = odds ratio; CI = confidence interval.

*The positive axillary LN was considered when one or more findings were noted as follows: cortical thickness>3 mm, eccentric cortical thickening, loss of fatty hilum, and round or lobulated nodal shape at MR imaging.

### Diagnostic Performance of Restaging Imaging for Advanced Nodal Disease

**Figure 1:**
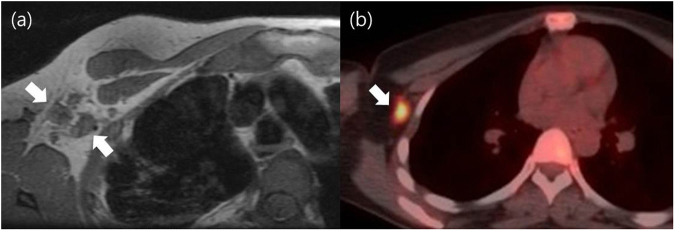
Transverse T1-weighted breast MR images (**a**) and axial PET/CT images (**b**) after neoadjuvant chemotherapy in 34-year old woman with invasive ductal carcinoma in her right breast. Breast MR image shows four suspicious nodes (arrows indicate two suspicious nodes among four) and PET/CT image shows FDG uptake (arrows) with a maximum standardized uptake value of 9.2. Axillary lymph node dissection revealed nine lymph node metastases (ypN2).

The optimal cut-off values for advanced ALN metastases were ≥2 positive ALNs and >7.7 mm SD of the largest ALN at restaging MR imaging and >1.1 nodal SUVmax at restaging PET/CT (Fig. 1). Table 5 summarizes the diagnostic performances of restaging MR imaging and PET/CT for the diagnosis of advanced ALN metastases. The NPV for excluding advanced ALN metastases was 97.6%, 94.4%, and 100.0% for restaging MR imaging, PET/CT, and MR imaging plus PET/CT, respectively. For restaging MR imaging, restaging PET/CT, and restaging MR imaging plus PET/CT, the sensitivity was 88.3%, 58.3%, and 100.0%, while the AUC was 0.792 (95% CI, 0.703 to 0.864), 0.729 (95% CI, 0.635 to 0.810), and 0.849 (95% CI, 0.7674 to 0.911), respectively. The AUC and sensitivity of restaging MR imaging plus PET/CT was higher than that of each restaging MR imaging and PET/CT; however, the differences were not statistically significant (AUC, *P* = 0.318 and *P* = 0.119, respectively; sensitivity, *P* = 0.500 and 0.063, respectively).

**Table 5:** Diagnostic Performance of Restaging MR Imaging and PET/CT after Neoadjuvant Chemotherapy for Prediction of Advanced ALN metastases.

Imaging modalities	AUC	Sensitivity	Specificity	PPV	NPV
MR imaging	0.792	83.3%	75.0%	29.4%	97.3%
PET/CT	0.729	58.3%	87.5%	36.8%	94.4%
Combined MR imaging plus PET/CT	0.849	100.0%	69.8%	29.3%	100.0%

AUC = area under the receiver operating characteristic curve; PPV = positive predictive value; NPV = negative predictive value; MR imaging = magnetic resonance imaging; PET/CT = positron emission tomography/computed tomography.

## Discussion

There has been a growing interest in the prediction of the axillary tumor burden after NAC in recent years. Previous studies have suggested the axillary ultrasound as a modality for evaluating the nodal response after NAC^10,22^. Breast MR imaging is considered as the most accurate imaging modality for providing anatomical information and allows the inspection of the axilla and breast^17,23^. PET/CT as a modality to image tumor viability based on increased glycolysis also enables the localization of abnormal ALNs showing metabolic changes with anatomical information from CT^24,25^. Both modalities offer advantages over ultrasound for the visualization of the whole area of the axilla irrespective of the characteristics of the patient (e.g., obesity) or the experience of breast imager. Our study demonstrated that, in addition to evaluation of the primary tumor, the axillary findings of breast MR imaging and PET/CT can be helpful in predicting nodal status after NAC treatment.

For the diagnosis of residual ALN metastases, including both low and high burden of axillary tumor, our study demonstrated that positive HR status and higher (>0.9) nodal SUVmax on restaging PET/CT were significant predictors after NAC, but not the findings on breast MR imaging. It is well known that HR-positive breast cancers are less chemosensitive than HR-negative breast cancers, resulting in a low burden of axillary tumor even with negative imaging^26–28^. Using univariate analysis, You *et al*. also reported that HR-positive breast cancers were associated with a higher rate of incorrect diagnosis by ultrasound and breast MR imaging for nodal response after NAC^14^. Taken together, these results indicate that restaging MR imaging is limited in predicting the nodal response in patients with low burden of axillary tumor or HR-positive breast cancer after NAC.

However, we found that patients had a higher likelihood of advanced ALN metastases when they had higher (≥2) numbers of positive ALNs on restaging MR imaging after NAC. This finding suggests the potential utility of breast MR imaging at restaging after NAC for predicting advanced ALN metastases in patients with breast cancer. In previous studies, the authors have demonstrated several clinico-pathological factors associated with the nodal response after NAC^7,29,30^. However, those studies did not incorporate the imaging findings at restaging after NAC as predictors for nodal response. To our knowledge, this is the first report addressing factors that predict the high burden of the axillary tumor after NAC. Based on our multivariate analyses, restaging MR imaging provided the only predictor of advanced ALN metastases after NAC, while there were no significant clinico-pathological factors even in the univariate analysis.

With a growing interest in less-invasive approaches of axillary surgery, some studies attempted to exclude advanced ALN metastases. This approach allows the selection of patients with a greater likelihood of no or limited axillary tumor burden and provides them the opportunity to avoid ALND associated with lymphedema and paresthesias. Neal *et al*. reported that axillary ultrasound could exclude advanced nodal disease in 96% of patients with early breast cancer^4^. Hyun *et al*. reported that breast MR imaging could exclude advanced ALN metastases in 99.6% and 94.0% of no-NAC and NAC groups, respectively^15^. We also observed high NPVs for exclusion of advanced ALN metastases with restaging MR imaging and PET/CT, with 97.3% and 94.4%. Of note, with combined restaging MR imaging and PET/CT modalities, the NPV was perfect (100.0%). For patients who had a lower number (<2) of positive ALNs and smaller (≤7.7 mm) SD of the largest LN on restaging breast MR imaging, and lower (≤1.1) nodal SUVmax on restaging PET/CT, ALND might be safely avoided. However, NPVs should be interpreted with caution in light of the intrinsic dependency of NPV on the prevalence of advanced ALN metastases.

For the determination of optimum cut-off values, we used ROC analysis and the current study found that the cut-off number (≥2) of positive ALNs on restaging MR for diagnosis of advanced ALN metastases is fewer than previous studies (≥4, analogous to the number of pathological nodal staging)^14,15^. It may be due to the fact that many metastatic ALNs can be normal-looking, especially when NAC treatment has been applied and therefore, the tumor burden of each LN is decreased^12^. Likewise, we found the optimum cut-off value for SUVmax to be 1.1, which is relatively low, perhaps due to the suppressed tumor glycolysis related to NAC treatment, occurring even in the presence of viable tumor cell^31^. We believe that cut-off values obtained from our ROC analyses can be used in predicting nodal status, thereby guiding less invasive axillary surgery.

Even though statistical significance was not obtained in the improvement of the diagnostic performance of combined restaging MR imaging and PET/CT compared with the use of either restaging MR imaging or PET/CT alone, we found that restaging MR imaging plus PET/CT improved the sensitivity compared with the use of either modality alone. Despite the slight decrease in specificity with restaging MR imaging plus PET/CT together, the overall diagnostic performance was increased. These observations may advocate the use of combined PET/MR imaging to evaluate axillary tumor burden. Taneja *et al*. reported a higher diagnostic accuracy of PET/MR in axillary nodal assessment compared with either MR imaging or PET alone^32^. In patients with cervical cancer and non-small cell lung cancer, improved detection of nodal metastases with PET/MR imaging was also reported^33,34^.

Several limitations of this study are worth noting. This was a retrospective study performed at a single institution. Although breast MR imaging and PET/CT for initial staging and restaging in consecutive patients with breast cancer undergoing NAC were routinely performed during this period, we did not control for a possible selection bias of patients who underwent breast MR imaging and PET/CT. Second, complete coverage of whole axillary region (level I-III) might not be possible with breast MR imaging, as the previous study have reported^5^. In our study, complete coverage of the field of view (FOV) for level I and level II area was generally achieved in all patients; however, artifacts or incomplete coverage was present for level III axillary area in some patients. Thus, a further study with a prospective design and a larger number of patients is needed. Third, as the cortex of ALNs usually became thinner after NAC, a partial volume effect that can cause underestimation in the observed SUVmax might have been introduced. Fourth, due to our retrospective design, the impact on decision for the type of axillary surgery was not direct or clear. Another caveat for this study is that not all patients underwent ALND for exact determination of nodal status. Although approximately 80% of the patients underwent ALND and 96% of patients with SLNB had ALNs negative for metastases, some patients still had advanced ALN metastases even with only one or two metastatic sentinel ALNs or with skipped metastases with negative sentinel ALNs.

To conclude, a higher (≥2) number of positive ALNs on restaging MR imaging was an independent predictor for advanced ALN metastases after NAC. The NPV for excluding advanced ALN metastases was high as 97.6%, 94.4%, and 100.0% for restaging MR imaging, PET/CT, and MR imaging plus PET/CT, respectively. Therefore, breast MR imaging and PET/CT at restaging have the potential to serve as tools to guide axillary surgery for a less-invasive approach in patients with invasive ductal carcinoma who are undergoing NAC.
